# Starvation of the bacterium *Vibrio atlanticus* induces simultaneous attacks on the dinoflagellate *Alexandrium pacificum*

**DOI:** 10.7554/eLife.107221

**Published:** 2026-06-12

**Authors:** Jean-Luc Rolland, Estelle Masseret, Mohamed Laabir, Guillaume Tetreau, Benjamin Gourbal, Anne Thebault, Eric Abadie, Alice Rodrigues-Stien, Carole Veckerle, Elodie Servanne-meunier, Delphine Destoumieux-Garzon, Arnaud Lagorce, Raphael Lami

**Affiliations:** 1 IHPE, Université Montpellier, CNRS, IFREMER, Université de Perpignan Via Domitia Montpellier France; 2 MARBEC, Université Montpellier, IRD, IFREMER, CNRS Montpellier France; 3 IHPE, Université Montpellier, CNRS, IFREMER, Université de Perpignan Via Domitia Perpignan France; 4 Agence nationale de sécurité sanitaire de l’alimentation, de l’environnement et du travail (ANSES), ANSES-DER 27-31 Maisons-Alfort France; 5 IFREMER, 9 rue Jean Bertho Le port, La Reunion France; 6 Sorbonne Université, CNRS, LBBM USR3579, Observatoire Océanologique de Banyuls sur Mer, Avenue Pierre Fabre Banyuls-sur-Mer France; 7 Sorbonne Université, CNRS, Bio2MAR, Observatoire Océanologique de Banyuls sur Mer, Avenue Pierre Fabre Banyuls-sur-Mer France; University of Lausanne Switzerland; University of Geneva Switzerland

**Keywords:** harmful algae, Interaction, dinoflagellate, vibrio, Predation, Interaction, Bacterium, Alga, Predation, Environment, Iron-related predation

## Abstract

Phytoplankton serve as a source of nutrients for bacteria in the marine environment. The interactions between algae and bacteria are known to include mutualism, commensalism, competition, or antagonism. This occurs in the microenvironment surrounding phytoplankton cells, the phycosphere, an interface rich in nutrients and organic molecules exuded by the cells. Here, based on *in situ* observations and on an *in vitro* interaction study, we report on a novel form of starvation-induced hunting that the cells of selected Vibrio species exert on dinoflagellates. The results showed that *Vibrio atlanticus* was capable of attacking and killing the dinoflagellate *Alexandrium pacificum* ACT03. Briefly, the observed mechanism of algal-killing consists of first, the ‘immobilization stage’ involving the secretion of algicidal metabolites that disrupt the flagella of the algae. In the ‘attack stage’, Vibrios simultaneously surround algal cells at high density for a brief period without invading them. Finally, the ‘killing stage’ in which the lysis and consumption of the dinoflagellates occur. By using a combination of biochemical, proteomic, molecular, and fluorescence microscopy approaches, we showed that this relationship is not related to the decomposition of algal organic matter, *Vibrio* quorum-sensing pathways, toxicity of the algae, or pathogenicity of the bacterium but is conditioned by nutrient stress, iron availability, and linked to the iron-vibrioferrin transport system of *V. atlanticus*. This is the first evidence of a new mechanism that could be involved in regulating *Alexandrium* spp. blooms and giving Vibrio a competitive advantage in obtaining nutrients from the environment. The interaction model we propose here suggests that Vibrio could play a role in regulating the proliferation of *Alexandrium* spp., giving it a competitive advantage in obtaining nutrients from the environment.

## Introduction

Harmful algal blooms (HABs) have experienced an increase in their occurrence, intensity, and geographical distribution on a global scale, resulting in adverse environmental, health, and socioeconomic impacts (Marampouti et al., 2021). HABs have a considerable impact on human health as a result of direct exposure to volatile toxins or by toxic seafood consumption (Burkholder et al., 2018). From an ecological point of view, the expansion of HABs can result in the erosion of biodiversity because they cause massive mortality of marine species and they are generally monospecific in nature (Chai et al., 2020). In coastal areas, understanding the biological interactions that control toxic algal blooms is therefore a major ecological challenge. Among HAB-causing organisms, a number of *Alexandrium* species have been placed on the list of invasive Mediterranean species. Among them, *Alexandrium pacificum* is a flagellated eukaryotic unicellular organism that together with *Alexandrium tamarense* and *Alexandrium fundyense* forms the ‘Alexandrium tamarense’ complex responsible for paralytic shellfish poisoning (PSP) worldwide (Hadjadji et al., 2020). Since 1998, *A. pacificum* (former *A. catenella*) was monitored by the French phytoplankton observation and monitoring network (Rephy) in the Thau lagoon (French Mediterranean) because it produces paralytic shellfish toxins (PSTs) resulting in PSP syndrome.

Laanaia (Laanaia et al., 2013) showed that in Thau lagoon, a water temperature around 20 °C for several days and organic and inorganic nutrients in sufficient concentrations are parameters favoring the development of *A. pacificum*, whose massive blooms occur in autumn. Algal blooms are seasonal events resulting in a rapid increase in the concentration of a species of algae in an aquatic environment. Depending on the species of phytoplankton, tolerance to physicochemical parameters varies, which influences when blooms occur (Rijal Leblad et al., 2020). Interestingly, although the collapse of phytoplankton blooms has been previously attributed to viruses (Pal et al., 2020), some ecological studies have suggested an important role of algicidal bacteria (Su et al., 2007; Wang et al., 2010). Among them, several are belonging to the Vibrio genus (Li et al., 2014; Wang et al., 2020).

Vibrio (class γ-proteobacteria) are common microorganisms in marine systems worldwide (Baker-Austin et al., 2017; Mavian et al., 2020), where they are important components of the food chain, particularly in biodegradation, nutrient regeneration, and biogeochemical cycles (Oberbeckmann et al., 2012). *Vibrio* is one of the most studied bacterial taxa due to their ubiquity in coastal marine systems and their capacity to cause infections in humans and animals, leading sometimes to epizootic or zoonotic epidemics (Roux et al., 2015; Mavian et al., 2020). *Vibrio* are extremely adaptable to their environment (Johnson, 2013). The main factors influencing their occurrence and distribution in water are temperature, salinity, nutrient availability (Wang et al., 2020), multiple strategies such as biofilm formation on biotic and abiotic surfaces (Espinoza-Vergara et al., 2020), or interactions with a multitude of other organisms such as eukaryotic predators (Drebes Dörr and Blokesch, 2020) or plankton (Lopez-Joven et al., 2018) are used by *Vibrio* in the environment. There is also evidence that global climate change has increased *Vibrio*-associated illnesses affecting humans and animals (Brumfield et al., 2021; Muhling et al., 2017). However, the drivers and dynamics of *Vibrio* survival and propagation in the marine environment are not yet fully understood.

A substantial number of research articles have highlighted the potential of γ-proteobacteria to exert algicidal activity against dinoflagellates, supporting the hypothesis that γ-proteobacteria such as *Vibrio* play a role in the control of algal blooms *in situ* (Coyne et al., 2022). However, the mechanisms behind *Vibrio*-driven algal lysis in the environment remain to be elucidated. Particularly, it is unclear how in the water column, algicidal compounds secreted by bacteria can concentrate around the algae to exert their lytic effect.

This study aims to describe observations made in the natural environment between *Vibrio* bacteria and *Alexandrium* algal blooms, and to determine *in vitro* the main factors involved in this relationship. Using a combination of biochemical, proteomic, molecular, and fluorescence microscopy approaches, we explored the role in algal toxicity, bacterial pathogenicity, and the quorum-sensing (QS) pathway on this relationship and showed the important role of nutrient stress and the iron uptake pathway in this unique *Vibrio*/*Alexandrium* interaction.

## Methods

### Quantification of *Alexandrium* algae and Vibrio bacteria in the environment by qPCR

Seawater samples were collected in the Thau Lagoon (southern France, a shallow Mediterranean ecosystem open to the sea Abadie et al., 1999), during spring and autumn 2015. Briefly, samples were collected from the subsurface (–50 cm) near an oyster table at a phytoplankton surveillance site (part of the REPHY network, N 43°26.058’ and E 003°39.878’). Once a week during spring and autumn 2015, during field sampling campaigns, 20 L of water was filtered on board through a 180-μm-pore-size nylon membrane. At the laboratory, according to Lopez-Joven et al., 2018 seawater was fractionated into two size classes as follows: 2 L of the above filtrate was filtered through a 0.8-μm-pore-size polycarbonate Whatman Nuclepore membrane to obtain organisms in the 0.8–180 μm range corresponding to plankton-associated *Vibrio* and living *Alexandrium* forms. Then, the filtrate from the 0.8 µm membrane was filtered again through a 0.2-μm-pore-size polycarbonate Whatman Nuclepore membrane until the membrane was saturated. *Alexandrium* cells, ranging from 25 to 40 µm, belong to the microphytoplankton and are therefore retained in the 0.8–180 µm fraction. Any *Vibrio* cells potentially associated with or attached to *Alexandrium* cells will also be retained in this fraction. *Vibrio* cells are approximately 0.5–0.8 µm thick. The fraction between 0.2 and 0.8 µm therefore includes the free-living *Vibrio*. The bacterial population collected on 0.8-µm-pore-size filters was designated the particle-associated community, and the population on 0.2-µm-pore-size filters was designated as the free-living community. Membranes (in triplicate) were then conserved in 500 µL of 100% EtOH at –20 °C. Environmental DNA (eDNA) was extracted from the MF Millipore membrane using the Macherey-Nagel NucleoSpin Tissue Kit and resuspended in 100 µL of water. The samples were then stored at –20 °C after eDNA quantity and purity were assessed using a NanoDrop system (NanoDrop Technologies, Wilmington, DE, USA). PCR amplification reactions were done on a Roche LightCycler 480 Real-Time thermocycler (qPHD platform, University of Montpellier, France) using specific primer pairs (Table 3). Typically, the reactions contained 1 µL of template DNA (the DNA concentration for all samples varied from 1 to 40 μg mL^−1^), 0.5 µL of each primer (3.33 µM), and 4 µL of reaction mixture (SYBR Green Master Mix) in a total volume of 6 μL. The reaction parameters were as follows: 5 min at 95 °C (initial denaturation) and 40 cycles of 10 s at 95 °C (denaturation), 10 s at the corresponding hybridization temperature (Table 3), and 10 s at 72 °C (elongation). Melting curve profiles were generated by increasing the temperature from 65°C to 95°C at 0.5 °C s^–1^. Amplification products were analyzed using LightCycler software (Roche Diagnostics). *Vibrio* spp. and *A. pacificum* and *A. tamarense* were quantified by constructing calibration curves based on DNA from the *Vibrio atlanticus* LGP32 reference strain (former *V. tasmaniensis* LGP32) and from the *A. pacificum* reference strain (ACT03: *A. catenella* strain isolated from Thau in 2003) and the *A. tamarense* reference strain (ATT07: *A. tamarense* isolated from Thau in 2007) (not shown).

### Strains and growth conditions

*Vibrio* strains. Wild-type and isogenic mutants of *V. atlanticus* LGP32 (Table 1) were used in this study. Deletion-mutants included Δ*luxR*, Δ*luxS*, Δ*luxM,* and Δ*pvuB* isogenic strains. The Δ*pvuB* mutant was constructed here by allelic exchange as described previously by Le Roux (Le Roux et al., 2007). We also used *V. atlanticus* LGP32 carrying the pSW3654T-GFP plasmid (Le Roux et al., 2007), hereafter referred to as *V. atlanticus* LGP32-GFP. Bacterial strains were grown at 22 ± 1°C in Zobell medium (0.38 µM iron (III)). When needed, 25 μg mL^–1^ chloramphenicol (Cm) was added to cultures of *V. atlanticus* LGP32-GFP (Le Roux et al., 2007).

**Table 1.** Ability of *Vibrio* strains to attack and to lyse *Alexandrium pacificum*. ND: not determined.

Vibrio species	Strains	Virulence for fish or invertebrates	References	Attack*A. pacificum*	Lyse*A. pacificum*	
*Vibrio atlanticus* LGP32	WT	Yes	Gay et al., 2004	+	+	
*//*	WT +pSW3654T-GFP	Yes	Le Roux et al., 2007	+	+	
*//*	ΔLuxM	ND	Ifremer Institute, France	+	+	
*//*	ΔLuxS	ND	//	+	+	
*//*	ΔLuxR	ND	//	+	+	
*//*	ΔPvuB	ND	This work	-	+	
*Vibrio tasmaniensis*	J5-9	Yes	Lemire et al., 2015	+	+	
*//*	LMG20012^T^	No	Thompson et al., 2003	+	+	
*Vibrio crassostreae*	J2.9	Yes	Lemire et al., 2015	+	+	
*//*	J2-8	No	//	+	+	
*Vibrio fischeri*	ES114	ND	Mandel et al., 2008	+	+	
*Vibrio harveyi*	ATCC14126	Yes	Liu et al., 1996	+	+	
*Vibrio aestuarianus*	janv-32	Yes	Labreuche et al., 2010	+	+	
						

#### Phytoplankton strains

Non-axenic phytoplankton species (Table 2) were grown in batch culture in enriched natural sea water (ENSW, 6.55 µM iron (III)) with a salinity of 36 practical salinity units (PSU) at 22 ± 1°C under cool white fluorescent illumination (100 µmol photons m^–2^ s^–1^) and a 12 hr:12 hr light:dark cycle (Harrison et al., 1980). The algae were used for experiments in their exponential growth phase.

**Table 2.** Ability of *V. atlanticus* LGP32 to degrade flagella, attack, and lyse the targeted dinoflagellates spp. commonly found in the Mediterranean Sea. ND not determined.

Dinoflagellates species	Strains	Toxicity for human	References	Flagella degraded	Cells Attacked	Cells Lysed
Alexandrium pacificum	ACT03, Thau, France	Yes	Laabir et al., 2011	+	+	+
Alexandrium catenella	Bizerte, Tunisia	Yes	Fertouna-Bellakhal et al., 2015	+	+	+
//	F3-9F, Tarragona, Spain	ND	//	+	+	+
//	C10-5, Annaba, Algeria	Yes	Hadjadji et al., 2020	+	+	+
Alexandrium tamarense	ATT07, Thau, France	No	Rolland et al., 2012	+	+	+
Alexandrium spp.	Golf of Tunis, Tunisia	ND	Algal collection University of Montpellier, France	+	+	+
//	Bizerte, Tunisia	ND	//	+	+	+
//	Mediterranean coast, Morocco	ND	//	+	+	+
Prorocentrum lima	PLBZT14, Bizerte, Tunisia	Yes	Ben-Gharbia et al., 2016	-	-	-
Coolia monotis	CMBZT14, Bizerte, Tunisia	ND	//	-	-	-
Vulcanodinium rugosum	IFR-VRU-01, Ingril, France	Yes	Abadie et al., 2015	-	-	-
Karenia selliformis	Golf of Gabes, Tunisia	ND	Algal collection University of Montpellier, France	-	-	-
Scripsiella trochoidae	Mellah Lagoon, Algeria	-	//	-	-	-
Gyrodinium impudicum	Golf of Tunis, Tunisia	-	//	-	-	-
Amphidium carterae	SAMS, Scotland	ND	SAMS laboratory, Scotland	-	-	-
Gymnodinium catenatum	M'diq Bay, Morocco	+	Rijal Leblad et al., 2020	+	+	+

### Co-culture assay

For each tested phytoplankton species (Table 2), 2x10^4^ cells harvested in their exponential growth phase (doubling time between 5 and 7 days) were placed in 20 mL of ENSW medium in a 50 mL suspension culture flask (Cellstar PS, Greiner bio-one). After incubation for 24 hr at 22 ± 1°C under cool white fluorescent illumination, 40 µL of *Vibrio* strains (Table 1) grown for 12, 36, 60, and 156 hr in Zobell medium or in Zobell medium supplemented with FeCl_3_ (6 µM iron (III)) or with boron (H_3_BO_3;_ 0.47 mM) or the corresponding culture supernatant were added to the phytoplankton cells (Table 2). After incubation at 22 ± 1°C for 0, 15, 30, 45, and 60 min under cool white fluorescent lights, living, non-swimming, attacked, and lysed phytoplankton cells were counted in a sedimentation chamber under an inverted microscope. The number of lysed cells corresponded to phytoplankton cells showing disrupted membranes. Non-swimming algae were not counted as lysed cells. For *Vibrio* analysis, 100 µL of a 1:10 serial dilution mixture in ENSW (from 10^–2^ to 10^–10^) was plated on Vibrio Selective TCBS (thiosulfate-citrate-bile salts-sucrose) agar (in triplicate). After incubation for 24 hr at 22 ± 1°C, the number of living *Vibrio* cells was determined by counting colony-forming units (CFUs). The data come from three independent experiments using independent phytoplankton cultures and independent bacterial cultures.

### Microscope observations

The dynamic of the interaction between *A. pacificum* ACT03 isolated from the French Thau Lagoon, south of France (Laabir et al., 2011) and *V. atlanticus* LGP32, which is an oyster pathogen isolated from the French Atlantic coast (Gay et al., 2004) and present in the Thau Lagoon (Lopez-Joven et al., 2018) was surveyed. As the *A. pacificum* ACT03 strain (Table 2) used in the study is not axenic, there is potential for bacteria other than *V. atlanticus* LGP32 to be present in the experiments. To elucidate the interaction without thoroughly accounting for the non-axenic cells, interaction was observed under a Zeiss Axio upright fluorescence microscope equipped with an AxioCamMRm 2 digital microscope camera using *V. atlanticus* LGP32 tagged with green fluorescent protein (GFP). Lasers were used at excitation wavelength (λex) 488 nm for GFP (emission wavelengths (λem): 505–530 nm) and λex 532 nm for plankton chloroplasts (λem 560–630 nm). Images were taken sequentially to avoid cross-contamination between fluorochromes. Sequences of images were merged during the *Vibrio*-*Alexandrium* interaction using ZEN 2012 (blue edition) software. Interaction events between *Vibrio* strains and phytoplankton strains were also observed under a Leica TCS SPE confocal laser scanning system connected to a Leica DM 2500 upright microscope camera (Montpellier RIO Imaging Platform, University of Montpellier, France).

### Comparative proteomic analysis

#### *Vibrio* sampling and protein extraction

*V. atlanticus* LGP32 was grown for 60 hr at 22 °C in artificial seawater (high nutrient stress, cond. 1) or 12 hr at 22 °C in Zobell media (no nutrient stress, cond. 2). After 10 min centrifugation at 8000 rpm, crude protein extracts of *V. atlanticus* LGP32 in each culture condition (triplicates) were obtained by sonication on ice at 20% amplitude for 20 s in 200 µL of ice-cold denaturing buffer (7 M urea, 2 M thiourea, 4% CHAPS in 30 mM Tris-HCl, pH 8.5) and clarified by centrifugation at 2000 x *g*, 15 min, 4 °C. The protein concentration of the supernatant was estimated using the 2D Quant Kit (Cytiva, MERCK) and samples were stored at –80 °C until use.

#### Two-dimensional gel electrophoresis (2D gel)

Protein extracts were individually analyzed on 2D gel electrophoresis (six gels per condition each corresponding to different biological replicates). To do so, 100 µg of proteins from each extract was added to rehydration buffer (7 M urea, 2 M thiourea, 4% CHAPS, 65 mM DTT) for a total volume of 350 µL. They were then individually loaded onto 17 cm isoelectric focusing strips (Bio-Rad) with a stabilized non-linear pH ranging from 3 to 10. Due to the high complexity of the protein profile in the acidic part (left) of the gel pH 3–10, we conducted additional ‘close-up’ analyses in gels using 17 cm isoelectric focusing strips (Bio-Rad) with a narrower, stabilized pH gradient ranging from 4 to 7. Strips were rehydrated passively for 5 hr at 22 °C, followed by active rehydration for 14 hr under a 50 V current at 22 °C (to help large proteins enter the strips). Thereafter, isoelectrofocusing was carried out using the following program: 50 V for 1 hr, 250 V for 1 hr, 8000 V for 1 hr and a final step at 8000 V for a total of 140,000 V.hr with a slow ramping voltage (quadratic increasing voltage) at each step. Focused proteins were reduced by incubating the strip twice in equilibration buffer (1.5 M Tris, 6 M urea, 2% SDS, 30% glycerol; bromophenol blue, pH 8.8) containing DTT (130 mM) at 55 °C. Then, they were alkylated by incubation with equilibration buffer containing iodoacetamide (135 mM) on a rocking agitator (400 rpm) at room temperature protected from light. Proteins were also separated according to their molecular weight (second dimension) on 12% acrylamide/0.32% piperazine diacrylamide gels run at 25 mA per gel for 30 min followed by 75 mA per gel for 8 hr using a Protean II XL system (Bio-Rad). Gels were stained using an MS-compatible silver staining protocol and scanned using a ChemiDoc MP Imaging System (Bio-Rad) associated with Image Lab software version 4.0.1 (Bio-Rad).

#### Comparative bioinformatics analysis of 2D gels

Twelve gels (six per condition) were selected for comparative analysis on PD-Quest v. 7.4.0 (Bio-Rad) to identify changes in protein abundance between the proteomic profiles of *V. atlanticus* LGP32 cultured in contrasting nutrient conditions (ENSW/Zobell). Spots whose mean intensity across six replicates per strain was two times higher or lower than those from the other strain, with a p<0.01 (Mann-Whitney U-test), were considered significantly different in terms of abundance between the two conditions (quantitative difference). Differentially represented spots were then excised from the gels, destained, trypsin-digested, and the obtained peptides were identified by tandem mass spectrometry (MS-MS) using the PISSARO platform facility (University of Rouen, France). To identify protein(s) present in each spot, the obtained peptides were compared with *V. atlanticus* LGP32 reference genome (https://vibrio.biocyc.org/). The genes whose peptides matched strongly were retrieved and used for a BLASTx query against non-redundant databases to determine the protein identity of the best match. A gene was considered as strongly matched when at least two peptides matched the sequence with a coverage of >6%. Their theoretical isoelectric point (pI) and molecular weights were also calculated using the Expasy server (https://www.expasy.org/) to compare them with the location of the spot on the gel. Altogether, these complementary analyses made it possible to characterize the protein identity of each spot with confidence.

### Gene expression analysis

#### *Vibrio* sampling and RNA extraction

*V. atlanticus* LGP32 was grown in Zobell media for 36 hr and 60 hr at 22 °C (decline phase of growth, nutrient stress) or for 12 hr at 22 °C (exponential growth phase, no nutrient stress). Total RNA was isolated from *V. atlanticus* LGP32 using the standard TRIzol method (Invitrogen Life Technologies SAS, Saint-Aubin, France) and then treated with DNase (Invitrogen) to eliminate genomic DNA contamination. After sodium acetate precipitation, the quantity and quality of the total RNA were determined using a NanoDrop spectrophotometer and agarose gel electrophoresis. Following heat denaturing (70 °C for 5 min), reverse transcription was performed using 1 μg of RNA prepared with 50 ng μL^–1^ oligo-(dT) 12–18 mer in a 20 μL reaction containing 1 mM dNTPs, 1 unit μL^–1^ RNAseOUT, and 200 units μL^–1^ Moloney murine leukemia virus reverse transcriptase (M-MLV RT) in reverse transcriptase buffer, according to the manufacturer’s instructions (Invitrogen Life Technologies SAS, Saint-Aubin, France).

#### PCR amplification

Amplification reactions were analysed using a Roche LightCycler 480 Real-Time thermocycler (Bio-Environnement platform, University of Perpignan, France). In this study, several PCR primer pairs were designed using Primer3 software (optimal primer size: 20 bases; Tm: 60 °C; primer GC%: 50; 2GC clamp and product size range: 150–200 bp) and calibrated with *V. atlanticus* LGP32 genomic DNA (Table 1). To determine the qPCR efficiency of each primer pair used, standard curves were generated using seven serial dilutions of genomic DNA (10^–2^, 10^–3^, 10^–4^, 10^–5^, 10^–7^, and 10^–8^; not shown); the qPCR efficiencies of the tested genes varied between 1.85 and 2.08 (Table 3). For gene expression, reverse transcription was performed with 1 µg of total RNA using random hexamers and SuperScript IV reverse transcriptase (Invitrogen). The total qPCR reaction volume was 10 μL and consisted of 5 μL of cDNA (diluted 1:5), 2.5 μL of SensiFAST SYBR No-ROX Mix (Bioline), and 100 nM or 300 nM PCR primer pair (Table 3). The reaction parameters were as follows: 2 min at 95 °C (initial denaturation) and 40 cycles of 5 s at 95 °C (denaturation), 10 s at 59 °C (annealing), and 20 s at 72 °C (elongation). The specificity of each PCR was checked by measuring fluorescent signals during melting curve analysis (PCR product heated from 65°C to 95°C continuously and slowly at 0.1 °C s^−1^). Relative expression was calculated by normalization to the expression of two constitutively expressed housekeeping genes, namely, 6PKF (VS_2913) and CcmC (VS_0852), using the delta-delta threshold cycle (ΔΔCt) method (Pfaffl, 2001).

**Table 3.** Oligonucleotide sequences of primers used for RNA expression analysis.

Species	genes	Primer Sequences	Tm (°C)	Efficiency	References
*Alexandrium pacificum* (ACT03)	18S – 28S rRNA ITS region	TGATATTGTGGGCAACTGTAA	54		Genovesi et al., 2011
		AACATCTGTTAGCTCACGGAA			
*Alexandrium tamarense* (ATT07)	18S – 28S rRNA ITS region	TGGTAATTCTTCATTGATTACAATG	54		//
		AACATCTGTTAGCTCACGGAA			
*Vibrios* spp.	16 S	CGGTGAAATGCGTAGAGAT	62		Kita-Tsukamoto et al., 1993
		TTACTAGCGATTCCGAGTTC			
*Vibrio atlanticus* LGP32	LuxN (VS_II0260)	CACTTGCTAGTATCATCGC	60	1.92	This work
		ATCGAGTTAGCAAGAGCAC			
//	LuxM (VS_II0261)	TCCACTTATCACAAACAGG	60	1.91	***//***
		ACTGTACTTCCATTTGTCG			
//	LuxP (VS_II0355)	AAGTTCAGGATGAACCTATC	60	1.89	***//***
		CAAAGAGATACTTTGCTGAG			
//	LuxS (VS_2562)	ACTCTCGAGCACCTATACG	60	1.85	***//***
		GAAGGCGTACCAATCAAGC			
//	CqsS (VS_1725)	GACATCTATTGATGTTATGC	60	1.91	***//***
		TCACCCACTTCACGTAACTG			
//	PvsA (VS_II0355),Vibrioferrin biosynthesis protein	CAGAGCAAGAGCTAGAACC	59	1.91	***//***
		TCGTTGAGAACCTGACGAG			
//	PvuB (VS_II1126), ABC transporter vibrioferrin uptake FecB	TAGTGCAACCATGGGAATCG	57	2.01	***//***
		TAAACCGTACGTAGACGCTC			
//	PvuA2 (VS_II1127), Vibrioferrin receptor FecA	GGAGCTACAAGCATTCGTTC	57	2.08	***//***
		TTCGTCATATGGTCGCTTCG			
//	Housekeeping gene 1, CcmC (VS_0852)	ATTGCCGCCTTTATCGGTTT	60		Vanhove et al., 2016
		CAAGCACCCCACATTGGTTT			
//	Housekeeping gene 2, 6-phosphofructokinase (VS_2913)	GCCGTCACTGTGGTGACCTT	60		//
		TGCTTCTTGCCTTTCGCAAT			

### Detection of quorum-sensing signaling molecules

#### Vibrio culture

To detect the QS molecules (AI-2, AI-1, and CAI-1), *V. atlanticus* LGP32 was grown in Zobell media for 12 hr (exponential growth phase, control) and 60 hr (decline phase of growth, nutrient stress).

#### AI-2 analysis

Bioluminescence assay using the QS bioluminescent of *Vibrio campbellii* MM32 (*luxN*::Cm, *luxS*::Tn5Kan) was used to detect AI-2 molecules in culture supernatants. Briefly, *Vibrio* cultures were centrifuged at 17,000 x *g* for 10 min, and the resulting supernatants were filtered on 0.22 μm. Then 20 μL of the filtrates were mixed with 180 μL of *V. campbellii* MM32 diluted 1:5000 then incubated at 30 °C and 100 rpm. Luminescence and cell density (OD620) were collected in triplicate and analysed according to Tourneroche et al., 2019.

#### AI-1 and CAI-1 extraction and LC-MS analysis

Chemical analyses were conducted with a Q Exactive Focus Orbitrap System coupled to an Ultimate 3000 ultrahigh-performance liquid chromatography (UHPLC) system (Thermo Fisher Scientific) according to Rodrigues et al., 2022. Briefly, ethyl acetate (2 mL) was added into each culture (2 mL). This mixture was shaken overnight at room temperature (150 rpm). The two phases were then separated, and the aqueous phase was extracted once again. The two obtained organic phases were pooled, and the solvent was evaporated under vacuum. The crude extracts were dissolved in 500 µL LC-MS grade methanol for analysis. The experiments were performed using biological triplicates, each of which was analyzed in triplicate. Analyses of extracts and standards (3 μL injected) were performed in electrospray positive ionization mode in the 50–750 m/z range in centroid mode. The parameters were as follows: spray voltage: 3 kV; sheath flow rate: 75; aux gas pressure: 20; capillary temperature: 350 °C; heater temperature: 430 °C. The analysis was conducted in Full MS data-dependent MS2 mode (Discovery mode). Resolution was set to 70,000 in Full MS mode, and the AGC (automatic gain control) target was set to 1x10^6^. In MS2, resolution was 17,500, AGC target was set to 2x10^5^, isolation window was 0.4 m/z, and normalized collision energy was stepped to 15, 30, and 40 eV. The UHPLC column was a Phenomenex Luna Omega Polar C18 1.6 μm, 150x2.1 mm. The column temperature was set to 42 °C, and the flow rate was 0.4 mL min^–1^. The solvent system was a mixture of water (A) with increasing proportions of acetonitrile (B), with both solvents modified with 0.1% formic acid. The gradient was as follows: 1% B 3 min before injection, then from 1 to 15 min, a gradient increase of B up to 100% (curve 5), followed by 100% B for 5 min. The flow was injected into the mass spectrometer starting immediately after injection. All data were acquired and processed using FreeStyle 1.5 software (Thermo Fisher Scientific).

#### Chemicals and solvents

N-acyl-homoserine lactones (AHL) were obtained from Cayman Chemical (Ann Arbor, MI, USA). Stock solutions were obtained by dissolving standards in methanol or dichloromethane (C18-AHL) at a concentration of 1 mg mL^–1^ and stored at –80 °C. Standard solutions for UHPLC-high-resolution tandem mass spectrometry (HRMS) analyses were prepared by diluting each individual standard solution with methanol in order to obtain a concentration range from 2000–20 ng mL^–1^. LC-MS grade methanol, acetonitrile, and formic acid were purchased from Biosolve (Biosolve Chimie, Dieuze, France), analytical-grade ethyl acetate was obtained from Sigma-Aldrich. Pure water was obtained from Elga Purelab Flex System (Veolia LabWater STI, Antony, France).

### Nature of lytic compounds secreted by *V. atlanticus* LGP32

To determine the temperature-sensitivity of the lytic compounds secreted, *V. atlanticus* LGP32 grown for 60 hr in Zobell media at 22 °C was filtered through a 10 kDa membrane (Amicon Ultra-4 filter unit). The eluate containing molecules with MW below 10 kDa was then incubated in a water bath at 100 °C for 30 min. Boiled filtrates (0.1 mL) were subsequently used to inoculate *A. pacificum* (ACT03 strain) cultures, then lytic activity was observed under the Leica TCS SPE confocal laser scanning system. Zobell media with the same treatment was used as control.

### Statistical analysis

#### Environmental data

Statistical analyses were performed using R 3.6.3 software (R Development Core Team, 2024) using RStudio. The relationship between *Alexandrium* and *Vibrio* was explored separately in spring and autumn. We used a generalized linear model specifying a Gaussian family. For spring, the dataset for salinity and temperature was complete (14 periods of observation). An influential period was detected and removed from the dataset because no dead *Alexandrium* cells were observed. The effects of explanatory variables such as log10 (Vibrio +1), salinity, and temperature were centered, reduced, and tested as fixed effects with a linear relationship. Model selection was performed using the Akaike information criterion corrected for small sample size (AIC_c_). Models were considered different whenever the difference between their AIC_c_ value and the lowest AIC_c_ value (ΔAIC_c_) was lower than 2 (Burnham and Anderson, 2002). *Alexandrium* distribution and model residuals were checked for normal distribution assumptions (QQ plot and Shapiro-Wilk test). For autumn, the dataset was complete for 10 periods of observation. Salinity and temperature were missing for three periods. We explored the relationship between *Alexandrium* and *Vibrio* alone using the method detailed above for spring.

*In vitro* data. Statistical analyses were performed using one-way ANOVA (analyzed by pair) followed by Tukey’s test (Statistica 10.0 software, StatSoft, Maison-Alfort, France). *p<0.05, **p<0.01, ***p<0.001.

## Results

### Concomitant occurrence of *A. pacificum* ACT03*, A. tamarense* ATT07, and free-living *Vibrio* spp. in the Thau lagoon

**Figure 1.**
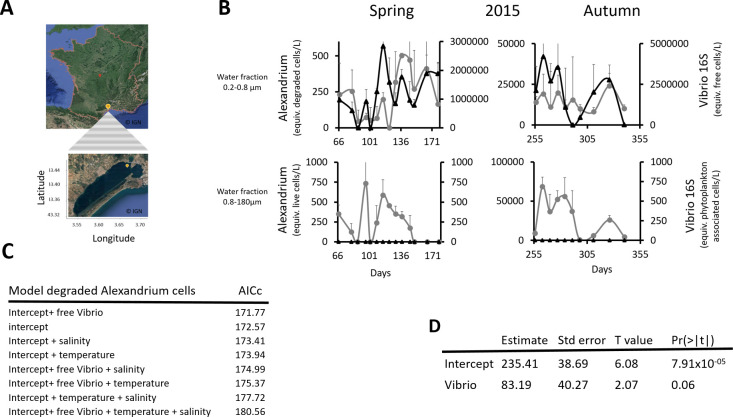
Dynamics of *Alexandrium* and *Vibrio* in the environment. (**A**) Location of the monitoring station in the Thau Lagoon (southern France). (**B**) Mean abundance (DNA equiv.) of *Vibrio* spp*.* (16 S) and *Alexandrium* spp*. (A. pacificum* ACT03*+A. tamarense* ATT07). *Vibrio* cells (black line with diamond dot) and degraded *Alexandrium* cells (grey line with round dot) were detected in the 0.2–0.8 μm fraction (free *Vibrio* fraction) in spring and autumn 2015. Living *Alexandrium* cells (grey line with round dot) but no plankton-associated *Vibrio* spp. (black line with diamond dot) were evident in the 0.8–180 μm in spring and autumn. (**C**) Results of the Akaike Information Criterion (AICc) test conducted to select a model for explaining the mean value of dead *Alexandrium* (degraded cells) in spring. (**D**) Wald test of the AICc model explaining the mean value of dead *Alexandrium* in spring by free *Vibrio*.

In the spring and autumn of 2015, in the Thau Lagoon (Figure 1A), we detected *Alexandrium* algae (*A. pacificum* ACT03 *and A. tamarense* ATT07, both alive and in degraded cell forms) and free-living *Vibrio*, but no plankton-associated *Vibrio* were observed (Figure 1B, Supplementary file 1). Using model selection based on AICc, we found no significant relationship between *Alexandrium* (*A. pacificum* ACT03*, A. tamarense*) and *Vibrio* spp. abundances in autumn. This result is consistent with *Vibrio*’s difficulty in growing at temperatures below 20 °C, as well as with the many environmental factors that can influence the dynamics of algae proliferation (Laanaia et al., 2013). Interestingly, in spring 2015, the mean densities of all *Alexandrium* cells (degraded and alive) and of free-living *Vibrio* were positively correlated. The lowest AICc was obtained with the model explaining degraded form of *Alexandrium* density based on the free *Vibrio* density (Figure 1C). Given that, this model is not so different from the model with only the intercept, but better than any other linear combination with other potentially interfering drivers, such as temperature and salinity (Figure 1D), we searched for evidence of a relationship between *Vibrio* and *Alexandrium* by studying their interaction *in vitro*.

### *V. atlanticus* LGP32 feeds on *A. pacificum* ACT03

**Figure 2.**
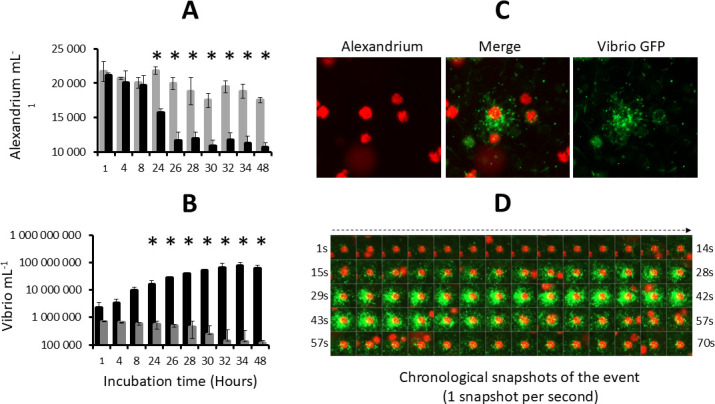
Incubation of *V.* *atlanticus* LGP32 and *Alexandrium pacificum* ACT03 in enriched natural seawater (ENSW). (**A**) *A. pacificum* ACT03 cultured alone (grey bar) and incubated with *V. atlanticus* LGP32 (black bar) in ENSW. (**B**) *V. atlanticus* LGP32 cultured alone (grey bar) and incubated with *A. pacificum* ACT03 (black bar) in ENSW. (**C**) Snapshot of the interaction between *V. atlanticus* LGP32-GFP cells (60 hour culture) and one cell of *A. pacificum* ACT03 taken at 8:00 of co-culture. (**D**) Chronological snapshots of the interaction (70 pictures, one per second). *V. atlanticus* LGP32 (small green cells) and *A. pacificum* ACT03 cell (large red cell). All experiments were done in triplicate. Asterisks indicate significant differences in a multiple comparison test (One-way ANOVA with post hoc Tukey test), *p≤0.05.

To investigate whether *Alexandrium* interacts with *Vibrio*, we incubated in Enriched Natural Sea Water (ENSW) *A. pacificum* ACT03 (2.0x10^4^ cells) with *V. atlanticus* LGP32 previously grown for 12 hr in Zobell media (initial concentration of 8.8x10^7^ cells mL^–1^). In interaction with *V. atlanticus* LGP32, *A. pacificum* ACT03 cell abundance decreased significantly from 2.1x10^4^ cells mL^–1^ after 1 hr of exposure to 1.1x10^4^ cells mL^–1^ after 48 hr of exposure (Figure 2A), while the *V. atlanticus* LGP32 concentration grew significantly after 26 hr of interaction, reaching a maximum peak density of 7.6x10^7^ CFU mL^–1^ at 34 hr (Figure 2B). In the control experiment where *A. pacificum* ACT03 was cultured alone in ENSW, the algal concentration remained stable over time (Figure 2A) and no bacteria were on the corresponding TCBS plates (Vibrio selective medium). In the control where *V. atlanticus* LGP32 was grown alone in ENSW, the bacterial concentration decreased from 7.0×10⁵ CFU/mL after 1 hr of incubation to 1.1×10⁵ CFU/mL after 48 hr of incubation (Figure 2B). These results show that the interaction between *V. atlanticus* LGP32 and *A. pacificum* ACT03 leads to a decline in the algal population and promotes the growth of *V. atlanticus* LGP32. This suggests that *V. atlanticus* LGP32 is able to feed on *A. pacificum* ACT03.

### *V. atlanticus* LGP32 performs attacks on *A. pacificum* ACT03

Epifluorescence microscopy observation of GFP-labeled *V. atlanticus* LGP32 (previously grown in Zobell medium) in interaction showed that *V. atlanticus* LGP32 cells are capable of simultaneously attacking *A. pacificum* ACT03 cells (Figure 2C and Video 1). The attacks were extremely rapid, with empty thecae (algal envelopes) observed in the medium after less than 60 s (Figure 2D and Video 2). During the attack, *V. atlanticus* LGP32 did not invade the algal cell but remained clustered on the cell surface (Figure 2C).

**Video 1.** Dynamics of *V. atlanticus* LGP32*-Alexandrium pacificum* ACT03 interaction. GFP-tagged *V. atlanticus* (small green cells); living *A. pacificum* (large red cells); lysed *A. pacificum* (large green cells) filmed under an epifluorescence microscope.

**Video 2.** Second-by-second timing of *V. atlanticus* LGP32 attacking *Alexandrium pacificum* ACT03. GFP-tagged *V. atlanticus* (small green cells); *A. pacificum* living cell (large red cells) filmed under an epifluorescence microscope.

### Attack of *A. pacificum* ACT03 is activated by *V. atlanticus* LGP32 starvation

**Figure 3.**
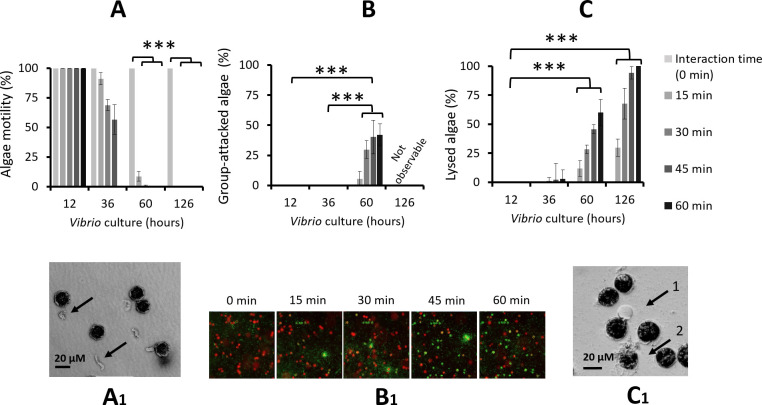
Role of *V. atlanticus* LGP32 starvation in the interspecific interaction process. Experiments were conducted by incubating *A. pacificum* ACT03 with *V. atlanticus* LGP32 previously grown for 12, 36, 60, and 126 h in Zobell medium. (**A**) Cumulative percentage of motile *A. pacificum* ACT03 cells. (**B**) Cumulative number of cells attacked by *V. atlanticus* LGP32 and (**C**) Cumulative cell lysis after 0, 15, 30, 45, and 60 min of interaction. Corresponding pictures showing (**A1**) Black arrows indicate unhooked and degraded flagellum from *A. pacificum* ACT03 flagellum, (**B1**) Chronological sequence of five snapshots showing *V. atlanticus* LGP32-GFP cells (60 hour culture) and *A. pacificum* ACT03 cells, during the first hour of their interaction. *V. atlanticus* LGP32 (small green cells), living *A. pacificum* ACT03 (large red cells), and dead *A. pacificum* ACT03 (large green cell). (**C1**) Black arrow 1 indicates vesicle formation on *A. pacificum* ACT03 cell and black arrow 2 indicates lysed *A. pacificum* ACT03 cell. All percentages were determined based on a minimum of 2000 cells of *A. pacificum* ACT03. All experiments were done in triplicate. Asterisks indicate significant differences in a multiple comparison test (One-way ANOVA with post hoc Tukey test), ***p≤0.001.

In 2002, Martin hypothesized that nutritional stress induces bacteria to lyse algae (Martin, 2002). To test this hypothesis, we monitored *V. atlanticus* LGP32 behavior in response to starvation (Figure 3). We observed that *V. atlanticus* LGP32 in exponential growth phase (12 hr of culture in Zobell medium, 8.8x10^7^ cells mL^–1^) did not interact with *A. pacificum* ACT03 cells for the first hour of contact (Figure 3A, B and C). In contrast, *V. atlanticus* LGP32 *in the* decline phase (*36 hr of* culture in Zobell medium, 1.3x10^7^ cells mL^–1^) induced a significant decrease in the number of motile algae cells by 8.9% after 15 min and by 43.3% after 60 min (Figure 3A). This phenomenon corresponded to the degradation and/or disruption of algal flagella (Video 3). The flagella no longer functioned correctly, which caused irregular swimming of the algae (Video 3, left cell). This was followed by a complete cessation of swimming. When the flagellum detached from the algae (Video 3, right cell), the attack occurred. With starved *V. atlanticus* LGP32 (60 hr of culture in Zobell medium, 0.6x10^7^ cells mL^–1^), algae immobilization was fast and significant (91.4% in 15 min, Figure 3A), and algae were attacked individually being targeted by *V. atlanticus* LGP32 cells (Video 4). The percentage of cells attacked and killed peaked at 30% after 15–30 min of contact (Figure 3B and B1) and then decreased. After 1 hr, attacks had stopped with approximately 40% of the algal cells still alive (Figure 3B1 and C). Although it remains unclear whether the attacks occur during a specific phase of growth, it is evident that the cells are already weakened before attack as they have all lost their flagella. An old-starved culture of *V. atlanticus* LGP32 (126 hr of culture in Zobell medium, <0.1 x 10^7^ cells mL^–1^) significantly immobilized *A. pacificum* ACT03 cells within a few minutes, with lysis occurring immediately (Figure 3A and C), making it impossible to detect attacks by *V. atlanticus* LGP32 (Figure 3B). The lysis phase corresponded to initial vesicle formation followed by the bursting of *A. pacificum* ACT03 cells (Figure 3C and C1 and Video 5). Importantly, *Vibrio* densities decreased with culture age, ruling out the possibility that the stronger predation observed in older cultures was driven by higher bacterial densities.

**Figure 3—figure supplement 1.**
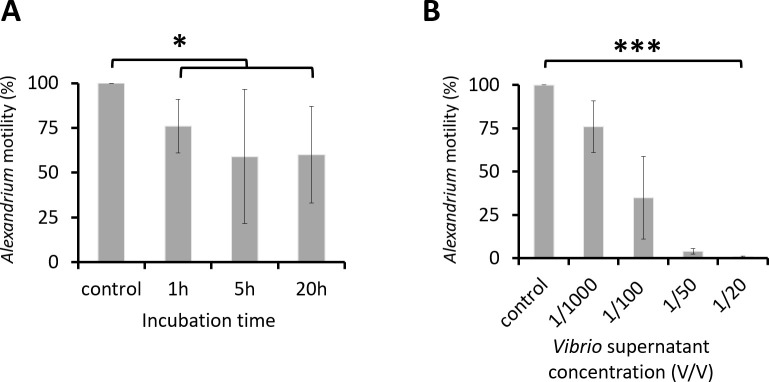
Time and dose-dependent effects of the *V.* *atlanticus* LGP32 culture supernatant on *A. pacificum* ACT03 motility. (**A**) A time dependence experiment was conducted by incubating *A. pacificum* ACT03 for 1, 5, or 20 hr with 1/1000 v/v (1 µL/mL) of culture supernatant from *V. atlanticus* LGP32 previously grown for 60 hr in Zobell culture media. (**B**) A dose-dependence experiment was conducted by incubating *A. pacificum* ACT03 for 1 hr with 1/1000 to 1/20 v/v (1–50 µL/mL) of culture supernatant from *V. atlanticus* previously grown for 60 hr in Zobell media. The percentage of motile *A. pacificum* ACT03 was determined after 1 hr of exposure. All percentages were determined based on a minimum of 2000 cells of *A. pacificum* ACT03. Error bars represent the standard deviation of the mean of three independent experiments. Asterisks indicate significant differences in a multiple comparison test (One-way ANOVA with post hoc Tukey test), *p≤0.05, ***p≤0.001.

**Video 3.** Degradation and disruption of *Alexandrium pacificum* ACT03 flagella. Effect of *Vibrio* supernatant on the first stage of the interaction filmed under a confocal microscope.

**Video 4.** Attacks of *V. atlanticus* LGP32 on target *Alexandrium pacificum* ACT03. This video, recorded under a confocal microscope, shows Vibrios simultaneously attacking a first immobilized *Alexandrium* cell, then moving on to attack a second cell without ever targeting the other cells present, suggesting active communication between the *Vibrio* bacteria. *V. atlanticus* LGP32 (small cells); *A. pacificum* ACT03 (large cells).

**Video 5.** Vesicle formation and bursting of an *Alexandrium pacificum* ACT03. Direct effect of *Vibrio* supernatant on *Alexandrium* after 126 hr of culture filmed under a confocal microscope.

We next tested whether this lytic effect was linked to *Vibrio* culture supernatant and mediated by thermostable molecule(s) secreted by *Vibrio* (Figure 3—figure supplement 1). The culture supernatant of starved culture of *V. atlanticus* LGP32 (60 hr) filtered through a 10 kDa membrane and then incubated at 100 °C for 30 min still possessed its lytic properties, indicating that the algicidal compounds produced by *V. atlanticus* LGP32 are small thermostable molecules unlikely to be lytic enzymes or lysins able to digest the algae cell. However, these experimental observations clearly show the key role of nutrient limitation in triggering the attack behavior and the secretion of lytic compounds of *V. atlanticus* LGP32.

### Attack occurs on *A. pacificum* ACT03 in exponential phase of growth

Here, we wondered whether the live/dead status of algae is important for *V. atlanticus* LGP32-mediated attacks targeting. To this end, *A. pacificum* ACT03 in exponential growth phase was first exposed for 30 min to the supernatant of a 126 hr culture of *V. atlanticus* LGP32, which induced lysis of 70% of the *A. pacificum* ACT03 cells (Figure 3C and C1 (arrow 2) and Video 4). Next, cells of *V. atlanticus* LGP32 from a 60 hour culture, capable of attacking *A. pacificum* ACT03 cells (Figure 3B), were added. For 1 hr of exposure, no attack was observed on the previously lysed algae. This result is similar to what is observed on the Video 1 with flash attacks only on immobilized, but not degraded *A. pacificum* ACT03 cells (red), and not on lysed cells (green). In addition, no attacks occurred on cells from an old *A. pacificum* ACT03 culture (1 month culture). Together, with the very short duration of attacks (Video 1), these results indicate that *V. atlanticus* LGP32 attacked exponentially growing cells of *A. pacificum* ACT03, but not decomposing algae, suggesting that this behavior is not just an opportunistic response of heterotrophic bacteria to organic substrates.

### Attack is independent of quorum sensing

**Figure 4.**
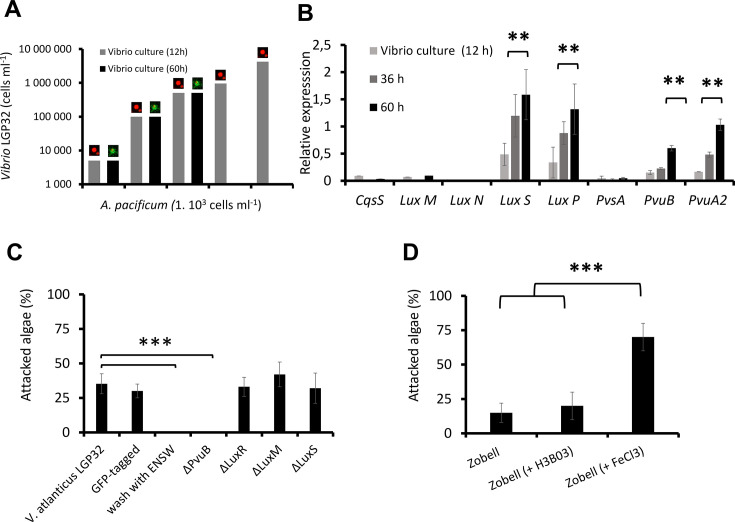
Role of quorum sensing and the vibrioferrin iron uptake pathway in the interaction process. (**A**) Effect of *V. atlanticus* LGP32 cell density on the attack process. *A. pacificum* ACT03 cells (1x10^3^ cells mL^–1^) were incubated with *V. atlanticus* LGP32 grown for 60 hr in Zobell medium at concentrations ranging from 5.10^3^ to 5 x 10^5^ cells mL^−1^ (black bars). For comparison, *A. pacificum* ACT03 incubated with *V. atlanticus* LGP32 grown for 12 hr in Zobell medium at concentrations ranging from 5x10^3^–4 x 10^6^ cells mL−1 (grey bars). The image on the bars indicates either unaffected algae (live red algae) or algae attacked by *Vibrio* (algae covered with green *Vibrio* cell) during the interaction (**B**) CqsS, luxM, luxN, luxS, and luxP quorum sensing and PvsA, PvuB, and PvuA2 vibrioferrin pathway genes expression in *V. atlanticus* LGP32 grown for 12, 36, and 60 hr in Zobell medium. (**C**) Effect of *V. atlanticus* LGP32 mutants on the attack process. Experiments were conducted by incubating *A. pacificum* ACT03 with *V. atlanticus* LGP32, *V. atlanticus* LGP32 tagged with GFP, *V. atlanticus* LGP32 washed with ENSW or *V. atlanticus* LGP32 mutant ΔPvuB, ΔluxM, ΔluxR, and ΔluxS previously grown 60 hr in Zobell media (control). The percentage of *A. pacificum* ACT03 attacked was determined during the first 30 min of exposure. (**D**) Effect of *V. atlanticus* LGP32 culture media composition on the attack process. Experiments were conducted by incubating *A. pacificum* ACT03 with *V. atlanticus* LGP32 grown 60 hr in Zobell media supplemented with boron (H_3_BO_3_) or FeCl_3_. The results were compared with an exposure to *V. atlanticus* LGP32 grown 60 hr in Zobell media. All percentages were determined based on a minimum of 2000 cells of *A. pacificum* ACT03. All experiments were done in triplicate. Asterisks indicate significant differences in a multiple comparison test (One-way ANOVA with post hoc Tukey test), **p≤0.01, ***p≤0.001.

Considering the simultaneous action of *V. atlanticus* LGP32 in attacks on *A. pacificum* ACT03, we tested whether the attack process depended on the key physiological mechanism that regulates many functions in marine microbial cells, QS (Lami, 2019; Papenfort and Bassler, 2016), a type of cell-cell communication. Although QS is a cell-density-dependent mechanism, our results showed no attack from a 12 hr culture of *V. atlanticus* LGP32 up to a concentration of 4x10^6^ Vibrio mL^–1^ (Figure 4A). Attacks were only observed with *V. atlanticus* LGP32 from a 60 hr culture at low concentration of 5x10^3^ Vibrio mL^–1^ to the highest concentration tested of 5x10^5^ Vibrio mL^–1^ (Figure 4A), consistent with the hypothesis that the attacks were independent of *Vibrio* density.

**Figure 4—figure supplement 1.**
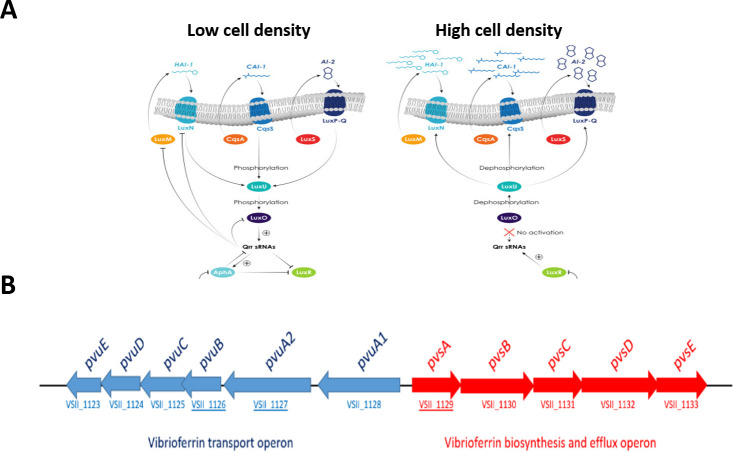
Quorum sensing (QS) and the vibrioferrin iron uptake pathway in *Vibrio*. (**A**) Putative QS pathways at low and high cell density in *Vibrio* according to Lami, 2019. (**B**) Genetic organization of the vibrioferrin utilization gene cluster on *V. atlanticus* LGP32 chromosome 2. The Pvu and Pvs operons are involved in the secretion and the transport of ferric vibrioferrin and biosynthesis of vibrioferrin, respectively. Arrows indicate the transcriptional directions of the genes. VSII1126, VSII1137, and VSII1129 corresponding to PvuB, PvuA2, and PvsA genes, respectively.

**Figure 4—figure supplement 2.**
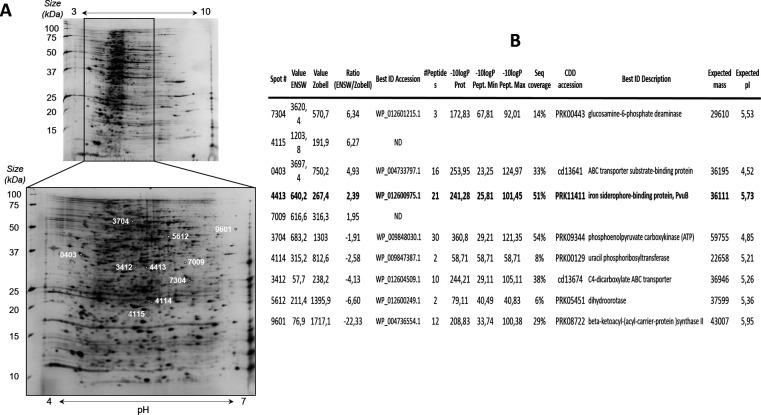
*V.* *atlanticus* LGP32 proteome analysis following nutrient stress. (**A**) Example of 2D gel, the numbers in white on the gel 4–7 correspond to the number and position of the protein spots analyzed. (**B**) Proteins identified by LC-MS/MS as differentially represented in the 2D gel comparative approach following nutrient stress. ND: Not determined; ENSW (artificial seawater).

The analysis of the expression of genes involved in the known QS pathways in *Vibrio* cell (Figure 4—figure supplement 1) highlighted that only the AI-2 pathway was induced during nutrient stress of *V. atlanticus* LGP32*,* because only the expression of the AI synthase (*LuxS*) and its receptor (*LuxP*) increased significantly (Figure 4B; ANOVA p<0.05). This was confirmed by a QS bioluminescence assay, which showed AI-2 molecules (unquantified) in the Zobell culture supernatant of *V. atlanticus* LGP32 after 60 hr of culture but not after 12 hr of culture and not in the ENSW supernatant of *V. atlanticus* LGP32 after 12 or 60 hr of culture. UHPLC-HRMS/MS provided no evidence of detectable HAI-1 and CAI-1 in any experiments.

Targeted mutagenesis of key genes involved in two of the three known QS pathways in vibrios (Figure 4—figure supplement 1), ΔluxM (HAI-1 production), ΔluxS (AI-2 production), and ΔluxR (main high-density QS regulator), did not result in any changes in the attack behavior of *V. atlanticus* LGP32 (Figure 4C). Combined with the absence of overexpression of the CqsS gene (inducible by CAI-1) involved in the last known QS pathway in *Vibrio* (Figure 4—figure supplement 1), these results indicated that the attack by *V. atlanticus* LGP32 is most likely unrelated to QS.

### *Attack* related to the availability of iron

The comparative analysis of the proteome of *V. atlanticus* LGP32 incubated 60 hr in artificial seawater (ENSW) versus *V. atlanticus* LGP32 grown 12 hr in Zobell nutrient-rich medium revealed 10 proteins that were differentially abundant under these two contrasting conditions (Figure 4—figure supplement 2). The two most down-regulated proteins correspond to be ß-ketoacyl-(acyl-carrier-protein) synthase II (–22-fold in ENSW compared to Zobell), a key regulator of bacterial fatty acid synthesis, and the dihydroorotase (–6.6-fold in ENSW compared to Zobell), an enzyme essential for pyrimidine biosynthesis and thus bacterial proliferation and growth. The low expression of these proteins in ENSW is consistent with *V. atlanticus* LGP32 nutritional starvation. The most up-regulated protein in starved *V. atlanticus* LGP32, with an increase of more than sixfold, was glucosamine-6-phosphate deaminase, an enzyme involved in bacterial energy metabolism probably necessary for its survival. Among the other up-regulated proteins, one was an iron siderophore-binding protein (Spot 4413, Figure 4—figure supplement 2) corresponding to the vibrioferrin outer membrane receptor PvuB, whose gene is part of the *pvu* operons involved in iron transport (Figure 4—figure supplement 1). Interestingly, the corresponding gene *pvuB* as well as the vibrioferrin membrane receptor gene *pvuA2* (Figure 4—figure supplement 1) were both significantly induced in *Vibrio* under nutrient stress (Figure 4B; ANOVA p<0.01) but not the one involved in the vibrioferrin biosynthesis, *pvsA* (Figure 4B). Remarkably, of the 10 proteins identified by proteomic analysis and eliminated by mutation, only elimination of PvuB prevented *V. atlanticus* from attacking *A. pacificum* ACT03 (Figure 4C; ANOVA p<0.001). In the absence of the *pvuB* gene, *V. atlanticus* LGP32 was unable to simultaneously attack *A. pacificum* ACT03. In addition, *V. atlanticus* LGP32 cells that had been washed with ENSW to remove their culture supernatant metabolites also failed to attack *A. pacificum* ACT03 (Figure 4C; ANOVA p<0.001), which is congruent with the hypothesis that attacks depend on the *V. atlanticus* LGP32 vibrioferrin transport system. Finally, attacks increased significantly when FeCl_3_ was added to the Vibrio culture medium (Figure 4D) but not with boron known to be capable of being transported by vibrioferrin (see Figure 4D). Taken together, those results are consistent with the hypothesis that attacks are regulated by iron.

### Attack is a *Vibrio* spp. behavior specific to *Alexandrium* spp

To evaluate the dinoflagellates specificity of the attack behavior, a selection of *Vibrio* spp. was co-cultured with a selection of dinoflagellate strains commonly found in the Mediterranean Sea. The results showed that, among the *Vibrio* spp. tested (pathogenic or not) all, when under nutrient stress, were able to secrete algicidal compounds, immobilize, attack and lyse *A. pacificum* ACT03 cells (Table 1) and no link to their pathogenicity for fish of invertebrates was observed (Table 1). Among the 16 dinoflagellates species tested, only *Alexandrium* spp. (non-toxic and PST producers) and *Gymnodinium catenatum* (PST producer) were immobilized, attacked, and lysed by *V. atlanticus* LGP32 (Table 2), but no link to PSTs was revealed (Table 2).

## Discussion

Predation is a widespread mode of interaction for survival in the natural world (Finke and Denno, 2004; Sinclair et al., 2003). Predatory bacteria are found in a wide variety of environments and are commonly described as feeding on other bacteria, although some cases of predation on microbial eukaryotes have also been hypothesized (Johnke et al., 2014; Pérez et al., 2016). Conceiving predators as free-living organisms that kill other organisms and feed on them, this study suggests that *Vibrio* engage in a novel form of predation in which they kill and feed on algae.

**Figure 5.**
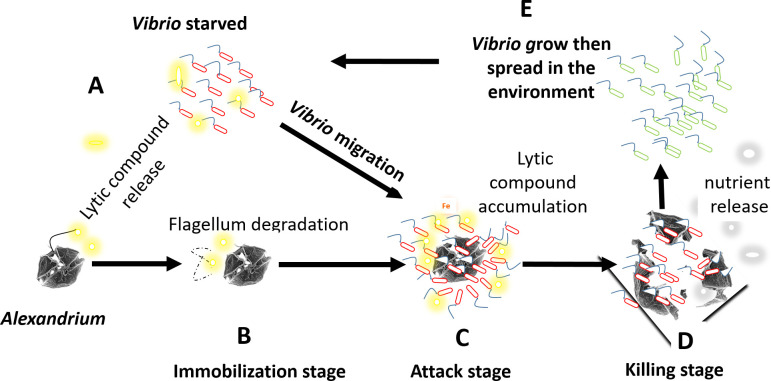
Schematic representation of a putative strategy developed by *Vibrio* spp. to feed on *Alexandrium* spp. and *G. catenatum* in the environment. (**A**) *Vibrio* in the environment when subjected to starvation secrete non-protein lytic compounds. (**B**) Some of these lytic compounds degrade the flagella, immobilizing the alga (immobilization stage). (**C**) Then *Vibrio* swims and clusters around its prey (attack stage). (**D**) Lytic compounds released by *Vibrio* were able to concentrate around the algal cells, thereby lysing the algae (killing stage). (**E**) Feeding on the released nutrients, *Vibrio* multiply and then spread in the environment. Yellow clouds: Lytic compound release by *Vibrio,* Grey clouds: Algal nutrients released upon lysis.

In fact, the strategy developed by *Vibrio* to kill algae may be reminiscent of strategies previously described in the prokaryotes (Johnke et al., 2014). As shown in Video 1, the interaction between *V. atlanticus* LGP32 and *A. pacificum* ACT03 proceeds in three stages (Figure 5). The first stage, the ***‘**immobilization stage*’, recalls the strategy used by *Streptomyces* to immobilize its prey (Kumbhar et al., 2014) based on the secretion of algicidal metabolites that disrupt the flagella. The second stage, the ‘attack stage’ corresponding to the physical contact between Vibrios and Alexandrium, is similar to the strategy used by *Myxococcus xanthus* and Lysobacter. These bacteria may require close proximity to their prey to cause lysis and utilize their biomass, although some can also kill prey at a distance through diffusible secretions (Martin, 2002; Genovesi et al., 2013; Pérez et al., 2016; Zhang et al., 2020). *V. atlanticus* LGP32 also surrounds *A. pacificum* ACT03 cells at high density for a very short time, but does not invade the algal cell. Visually, this phenomenon resembles bacteria clustering around lysed ciliate cells (Blackburn et al., 1998). The third stage, the ‘*killing stage*’, is similar to that of epibiotic bacterial predators, which induce the lysis of bacteria from the outside (Rashidan and Bird, 2001). Overall, these observations suggest that *V. atlanticus* LGP32 can exhibit a predatory behavior.

The attack behavior of *V. atlanticus* LGP32 was linked to iron absorption, as mutants with impaired iron absorption completely lost their ability to attack the algae. Iron is an essential element for growth in most organisms, including phytoplankton (Martin and Fitzwater, 1988) and bacteria (Neilands, 1981), and its concentration in seawater is known to be very low, with measurements in the open ocean surface at 0.2 nM and in deep waters at 0.6 nM (Millero, 1998).

Moreover, its low solubility in seawater limits its availability (Bruland et al., 1994; Wu and Luther, 1994). To acquire iron, bacteria have developed systems based on the secretion (and subsequent uptake) of iron-chelating siderophores to obtain this element from the environment (Amin et al., 2009a). Therefore, many *Vibrio* spp. produce a siderophore known as vibrioferrin, which is synthesized and secreted by proteins encoded by the *pvsABCDE* gene cluster (Figure 4—figure supplement 1). Given that boron is known for its role in regulating a global bacterial cellular response to phytoplankton and to bind to vibrioferrin (Romano et al., 2013; Weerasinghe et al., 2013), we tested its potential involvement in simultaneous *Vibrio* attacks. Compared to the Zobell control, no effect on the number of attacks was observed. For iron-vibrioferrin uptake, *Vibrio parahaemolyticus* uses a membrane siderophore receptor, called PvuA, which is coupled to an inner membrane ATP-binding cassette (ABC). This ABC transporter system, which is comprised of proteins encoded by the pvuABCDE gene cluster (Figure 4—figure supplement 1), is required for the transport of the siderophore across the inner membrane (Tanabe et al., 2003). Siderophores are not only iron carriers but also important regulators of virulence (Miethke and Marahiel, 2007) and mediators of bacterial interaction with phytoplankton (Amin et al., 2009b; Kramer et al., 2020). We showed here a pivotal role of iron in the interaction between *V. atlanticus* LGP32 and *A. pacificum* ACT03. This mirrors the mutualistic interaction observed between *G. catenatum* and *Marinobacter* (Amin et al., 2009b). In fact, in natural settings, the co-occurrence of *Marinobacter* and *G. catenatum* is suggested to depend on a mutually beneficial utilization of iron and carbon resources (Bolch et al., 2011). As in the present study, iron seems to play a key role in the interaction. Indeed, the labile iron released through the photolysis of ferric chelates with vibrioferrin provides a crucial iron source for phytoplankton, which need substantial amounts of iron to support carbon fixation through photosynthesis (Amin et al., 2009a; Yang et al., 2021). This fixed carbon, in turn, sustains the growth of both the phytoplankton and their associated bacterial counterparts (Amin et al., 2009b; Kramer et al., 2020). Interestingly, if a general nutrient deficiency causes attacks, iron supplementation increases this number of attacks (Figure 4D), suggesting the importance of iron absorption in the attack behavior. Future studies should determine whether a nutrient deficiency increases the iron absorption capacity of *Vibrio* bacteria and whether this could play a major role in the attack mechanism.

This study showed that QS is not involved in microbial attacks. Thus, *V. atlanticus* LGP32 mutants lacking the genes involved in known QS pathways exhibit the same phenotype as wild-type *V. atlanticus* LGP32, and *Vibrio* density does not induce attacks. However, *V. atlanticus* LGP32 produces AI-2 during attacks. QS and iron acquisition are sometimes interconnected in *Vibrio* (McRose et al., 2018). For example, in *Vibrio vulnificus*, the production of vulnibactin (a siderophore) is known to be controlled by AI-2 (Kim and Shin, 2011). Similarly, AI-2 could be involved in the production of vibrioferrin in *V. atlanticus* LGP32.

In the natural environment, associations between bacteria and algae have already been observed (Lopez-Joven et al., 2018; Miller et al., 2005; Rosales et al., 2022; Xu et al., 2022). We have shown here that algal attacks by *Vibrio* can be carried out *in vitro*. Environmental data collected in the Thau lagoon showed a correlation between the presence of *Alexandrium* and that of *Vibrio*, suggesting that such interactions could also occur in the marine environment. If that were the case, this behavior would provide an important ecological advantage to *Vibrio* to obtain nutrients in the environment, where *Alexandrium* spp. and *G. catenatum* form blooms.

With more than 30 species distributed all over the world (Anderson et al., 2012; Hallegraeff et al., 2021), *Alexandrium* spp. and *Gymnodinium* spp., considered as invasive species by the Delivering Alien Invasive Species Inventories for Europe (http://www.europe-aliens.org), could play an unexpected and important role in maintaining, structuring, and regulating *Vibrio* populations in the ecosystem. In turn, *Vibrio* could contribute to the regulation and control of their blooms.

To conclude, this study reveals the capacity of some *Vibrio* spp. to act as facultative predator-like bacteria that hunt specific algae. In the current context of climate change, which is favorable to their development, monitoring the invasive algae *Alexandrium* spp. and *G. catenatum* should be considered not only for their potent harmful effect on humans and animals, but also because they may represent a potential source of nutrients for the expansion of *Vibrio*, particularly pathogenic species (Lemire et al., 2015).

## Data Availability

Data generated or analysed during this study are included in the manuscript.

## Additional files

MDAR checklist

Supplementary file 1. Abundance of Alexandrium spp. and Vibrio spp. monitored by qPCR in the Thau Lagoon during spring and autumn 2015.
