# Supplementary material for: Starvation of the bacterium Vibrio atlanticus induces simultaneous attacks on the dinoflagellate Alexandrium pacificum
Source: eLife. 2026 Jun 12;14:RP107221. doi: 10.7554/eLife.107221 (PMC13263038; doi:10.7554/eLife.107221)
Supplement: Peer review history

# Editor report: eLife Assessment

**DOI:** 10.7554/eLife.107221.4.sa0

**Authors:** Sara Mitri

**Affiliations:** University of Lausanne Switzerland

**Keywords:** Convincing, Important

This **important** study **convincingly** shows that Vibrio bacteria act as predators of ecologically significant algae that contribute to harmful blooms in the lab and in their natural habitat, and that predation is induced by starvation. The authors suggest a working model that can be the basis for future work on this system. The study will be very impactful to those interested in the diversity of microbial predator-prey interactions and controlling toxic algal bloom.

# Referee report: Reviewer #1 (Public review)

**DOI:** 10.7554/eLife.107221.4.sa1

[Editors' note: this version has been assessed by the Reviewing Editor without further input from the original reviewers. We appreciate the revisions and the authors addressed all of the remaining minor concerns listed by the reviewers. We have no further suggestions for revision.]

Summary:

Rolland and colleagues investigated the interaction between Vibrio bacteria and Alexandrium algae. The authors found a correlation between the abundance of the two in the Thau Lagoon and observed in the laboratory that Vibrio grows to higher numbers in the presence of the algae than in monoculture. Timelapse imaging of Alexandrium in coculture with Vibrio enabled the authors to observe Vibrio bacteria in proximity to the algae and subsequent algae death. The authors further determine the mechanism of the interaction between the two and point out similarities between the observed phenotypes and predator prey behaviours across organisms.

Strengths:

The study combines field work with mechanistic studies in the laboratory and uses a wide array of techniques ranging from co-cultivation experiments to genetic engineering, microscopy and proteomics. Further, the authors test multiple Vibrio and Alexandria species and claim a wide spread of the observed phenotypes.

Comments on revisions:

I thank the authors for their additional work on the manuscript. My comments were addressed to my satisfaction.

# Referee report: Reviewer #2 (Public review)

**DOI:** 10.7554/eLife.107221.4.sa2

Goal summary:

The authors sought to (i) demonstrate correlations between the dynamics of the dinoflagellate Alexandrium pacificum and the bacterim Vibrio atlanticus in natural populations, (ii) demonstrate the occurrence of predation in laboratory experiments, (iii) demonstrate that predation is induced by predator starvation, and (iv) test for effects of quorum sensing and iron-uptake genes on the predation process.

Strengths include:

- Data indicating correlated dynamics in a natural environment that increase the motivation for study of *in vitro* interactions

- Experimental design allowing clear inference of predation based on population counts of both prey and predators in addition to microscopy-based evidence

- Supplementation of population-level data with molecular approaches to test hypotheses regarding possible involvement of quorum sensing and iron update in predation

Weaknesses include:

- A quantitative analysis of effects of manipulating V. atlanticus density on rates of predation would have been valuable

Appraisal:

The authors convincingly demonstrate that V. atlanticus can prey on A. pacificum, provide strongly suggestive evidence that such predation is induced by starvation and clearly demonstrate that both iron availability and correspondingly the presence of genes involved in iron uptake strongly influence the efficacy of predation.

Discussion of impact:

This paper will interest those interested in the diversity of forms of microbial predation and how microbial predatory behavior responds to environmental fluctuations. It will also interest those investigating bacteria-algae interactions and potential ecological controls of algal blooms. It may also interest researchers of microbial cooperation in light of the suggestion of communication between predator cells.

# Author comment: Author response

**DOI:** 10.7554/eLife.107221.4.sa3

**Authors:** Jean Luc Rolland, Estelle Masseret, Mohamed Laabir, Guillaume Tetreau, Benjamin Gourbal, Anne Thebault, Eric Abadie, Alice Rodrigues-Stien, Carole Veckerle, Elodie Servanne-meunier, Delphine Destoumieux-Garzon, LAGORCE LAGORCE, Raphael Lami

**Affiliations:** Marine Biodiversity Exploitation and Conservation Montpellier France; Marine Biodiversity Exploitation and Conservation Montpellier France; Marine Biodiversity Exploitation and Conservation Montpellier France; UMR 5244, IHPE, University of Perpignan Via Domitia Perpignan France; UMR 5244 IHPE CNRS Perpignan France; Agence Nationale de sécurité Sanitaire de l'Alimentation, de l'Environnement et du Travail Maison-Alfort France; Ifremer Le port, La Reunion France; Sorbonne Université Banyuls-sur-Mer France; Sorbonne Université Banyuls-sur-Mer France; UMR 5244, IHPE, University of Perpignan Via Domitia Perpignan France; UMR 5244, IHPE, University of Montpellier Montpellier France; Université de Perpignan Perpignan France; Sorbonne Université Banyuls-sur-Mer France

The following is the authors’ response to the previous reviews

**Public Reviews:**

**Reviewer #1 (Public review):**

Summary:

Rolland and colleagues investigated the interaction between Vibrio bacteria and Alexandrium algae. The authors found a correlation between the abundance of the two in the Thau Lagoon and observed in the laboratory that Vibrio grows to higher numbers in the presence of the algae than in monoculture. Timelapse imaging of Alexandrium in coculture with Vibrio enabled the authors to observe Vibrio bacteria in proximity to the algae and subsequent algae death. The authors further determine the mechanism of the interaction between the two and point out similarities between the observed phenotypes and predator prey behaviours across organisms.

Strengths:

The study combines field work with mechanistic studies in the laboratory and uses a wide array of techniques ranging from co-cultivation experiments to genetic engineering, microscopy and proteomics. Further, the authors test multiple Vibrio and Alexandria species and claim a wide spread of the observed phenotypes.

Comments on revisions:

I thank the authors for their additional work on the manuscript. My comments were addressed to my satisfaction.

Dear Reviewer #1, we thank you for your careful evaluation of our manuscript and for the time and effort you dedicated to this review. We are pleased that the revised version has addressed your concerns to your satisfaction.

**Reviewer #2 (Public review):**

Goal summary

The authors sought to (i) demonstrate correlations between the dynamics of the dinoflagellate Alexandrium pacificum and the bacterim Vibrio atlanticus in natural populations, (ii) demonstrate the occurrence of predation in laboratory experiments, (iii) demonstrate that predation is induced by predator starvation, and (iv) test for effects of quorum sensing and iron-uptake genes on the predation process.

Strengths include

- Data indicating correlated dynamics in a natural environment that increase the motivation for study of *in vitro* interactions

- Experimental design allowing clear inference of predation based on population counts of both prey and predators in addition to microscopy-based evidence

- Supplementation of population-level data with molecular approaches to test hypotheses regarding possible involvement of quorum sensing and iron update in predation

Weaknesses include

- A quantitative analysis of effects of manipulating V. atlanticus density on rates of predation would have been valuable

- Lack of clarity in some of the methodological descriptions

Appraisal

The authors convincingly demonstrate that V. atlanticus can prey on A. pacificum, provide strongly suggestive evidence that such predation is induced by starvation and clearly demonstrate that both iron availability and correspondingly the presence of genes involved in iron uptake strongly influence the efficacy of predation.

Discussion of impact

This paper will interest those interested in the diversity of forms of microbial predation and how microbial predatory behavior responds to environmental fluctuations. It will also interest those investigating bacteria-algae interactions and potential ecological controls of algal blooms. It may also interest researchers of microbial cooperation in light of the suggestion of communication between predator cells.

Dear Reviewer #2, we sincerely thank you for the time you devoted to this second review of our manuscript. We greatly appreciate your thoughtful comments, which helped us further improve the clarity and precision of the manuscript. All your additional recommendations have been carefully considered and addressed in the revised version and in our responses below.

**Recommendations for the authors:**

**Reviewer #2 (Recommendations for the authors):**

(2) The authors' reference to Fig. 4a did not address our concern about density potentially affecting the outcomes shown in Fig. 3. Fig. 4a does not provide any quantitative effects of manipulating Vibrio density. But the new density numbers the authors added in response to point (33) do seem to address our concern, because Vibrio densities become lower in the older cultures, excluding the possibility that the increased predation in older cultures might have been due higher Vibrio densities. We think this should be stated explicitly.

(33) See point (2) above. We think the authors should explicitly state in the text that the increased predation in older cultures was not due higher Vibrio densities in those older cultures, referring to their data.

As recommended by Reviewer#2, we added the sentence “Importantly, Vibrio densities decreased with culture age, ruling out the possibility that the stronger predation observed in older cultures was driven by higher bacterial densities” in the results section “Attack of *A. pacificum* ACT03 is activated by *V. atlanticus* LGP32 starvation.”

(45) Is it known that bacterial predators collectively feed more on other bacteria than on microbial eukaryotes in natural habitats? While this certainly seems most likely, it's stated as fact and so should either the statement should be supported with relevant citations or phrased as a likely hypothesis.

As suggested, we rephrased this sentence “Predatory bacteria are found in a wide variety of environments and are commonly described as feeding on other bacteria, although some cases of predation on microbial eukaryotes have also been hypothesized” in the discussion section.

(46) Perhaps "Conceiving predators as free-living organisms that kill other organisms and feed on them, this study suggest that Vibrios engage in a novel form of predation in which they kill and feed on algae."

The reference to 'developing' a predator behavior is not clear. What is meant by 'develop'? It seems unnecessary.

The use of italics when writing Vibrio is inconsistent.

We agree that the reference to “developing” a predatory behavior was unclear and unnecessary. We therefore revised the sentence as follows: “Conceiving predators as free-living organisms that kill other organisms and feed on them, this study suggests that Vibrio engages in a novel form of predation in which it kills and feeds on algae.” We also corrected the inconsistent use of italics for Vibrio throughout the manuscript.

(48) The authors might wish to revise this sentence, as although M. xanxthus does have contact-dependent killing mechanism, it is our understanding that both Lysobacter and myxobacteria can kill some prey at a distance with diffusible secretions.

The sentence “These bacteria must be in close proximity to their prey in order to cause lysis and utilize their biomass, regardless of the prey's species” was replaced by “These bacteria may require close proximity to their prey to cause lysis and utilize their biomass, although some can also kill prey at a distance through diffusible secretions”.

(50) Why not directly say 'predatory behavior?

We totally agree and have reworded the sentence.

Line by line feedback:

28 '...the phycosphere, an interface ...'

We agree and have revised the wording.

24 'In the attack stage, Vibrios...'

This sentence has been rephrased as recommended.

35 surrounds -> surround

The correction has been done.

36 The lysis is induced by the cells not by the 'stage'. We would rephrase to 'in which the lysis and consumption of the dinoflagellates occurs'

This sentence has been rephrased as recommended.

41 'a new mechanism that could to be involved' -> 'a new mechanism that could be involved ...'

The correction has been done.

61 forms

The correction has been done.

98 'the role...in'

The suggested correction has been performed.

103 'Qpcr' -> 'qPCR'

Thank you for spotting this typo. “Qpcr” was corrected to “qPCR” in the manuscript.

125 Misplaced punctuation

The punctuation was corrected.

152 The use of '.' vs 'x' to indicate multiplication when writing numbers is inconsistent. In some cases both are missing.

Numbers have been corrected throughout the manuscript.

231 I would rephrase 'poor nutrient stress' to 'little nutrient stress' or 'no nutrient stress'

The rephrasing was carried out as suggested.

310 R and used packages are not cited

We added the citation (R Core Team, 2024). Linear models, QQ plots (which are part of linear models), tests, and AICs are included in R by default and are credited to the R Core Team.

The sentence “Statistical analyses were performed using R 3.6.3 software” was replaced by “Statistical analyses were performed using R 3.6.3 software (R Core Team, 2024) using Rstudio”.

358 'are capable of simultaneously attacking'

The expression “are capable of simultaneously attacking” was revised in the manuscript to improve clarity and readability.

366 'exponential growth phase'

We have corrected the wording to “exponential growth phase” in the revised manuscript.

430 The large difference in incubation time between the sea-water vs nutrient-rich treatments and use of different media are unfortunate. These additional variables compromise the ability to directly ascribe observed differences to starvation.

We agree, the sentence “The comparative analysis of the proteome of *V. atlanticus* LGP32 incubated 60 h in artificial seawater (ENSW) versus *V. atlanticus* LGP32 grown 12 h in Zobell nutrient-rich medium revealed 10 proteins modulated by nutrient stress (Fig. S2)” was replaced by “The comparative analysis of the proteome of *V. atlanticus* LGP32 incubated 60 h in artificial seawater (ENSW) versus *V. atlanticus* LGP32 grown 12 h in Zobell nutrient-rich medium revealed 10 proteins that were differentially abundant under these two contrasting conditions (Fig. S2)”

443 Somewhat unclear sentence. I would rephrase this to "Remarkably, of the 10 proteins identified by proteomic analysis and eliminated by mutation, only elimination of PvuB prevented V. atlanticus from attacking A. pacificum ACT03."

To clarify this point, the sentence “Remarkably, among the 10 proteins identified by proteomic analysis only *V. atlanticus* LGP32 mutant lacking pvuB failed to attack *A. pacificum* ACT03 (Fig. 4C; ANOVA p <0.001)” was replaced by “Remarkably, of the 10 proteins identified by proteomic analysis and eliminated by mutation, only elimination of PvuB prevented *V. atlanticus* from attacking *A. pacificum* ACT03 (Fig. 4C; ANOVA p <0.001).”

445 'attack simultaneously' -> 'simultaneously attack'

The suggested modification has been done.

450 H3BO4 is written as Boron later, it would be good to call it boron here as well so that it is easier to make the connection for the reader.

We agree, we modified the manuscript and called it boron.

459 'no linked' -> 'no link'

The text was modified accordingly.

483 'which induces' -> 'which induce'

The correction has been made.

519 The use of Vibrio atlanticus and V. atlanticus is inconsistent within the text.

We have checked and modified the manuscript in accordance with the recommendations.

807-808 The use of the phrase 'Akaike information criterion (AICc) models' is confusing. Aren't these models just generalized linear models? It should be rephrased to make clear that the AICc is just a test that is used to select which model to use.

We clarified this point by revising Figure 1 legend. The sentences “(C) Result of Akaike information criterion (AICc) models tested to explain the mean value of degraded Alexandrium cells (dead cells) in spring. (D) Wald test of the AICc model attributing the mean value of degraded cells of Alexandrium in spring to free Vibrio “were replaced by “(C) Results of the Akaike Information Criterion (AICc) test conducted to select a model for explaining the mean value of dead Alexandrium (degraded cells) in spring. (D) Wald test of the AICc model explaining the mean value of dead Alexandrium in spring by free Vibrio”

827 The chronological sequence of snapshots is not very clear. Perhaps it would be clearer if pictures over a shorter timeframe were used to clearly show the gathering of the V. atlanticus cells near the algal cells.

To address this point, we removed the first and the last 14 seconds of the snapshots to clearly show the gathering of the *V. atlanticus* cells near the algal cells, and we added an arrow on Fig. 2D to indicate the chronological order.
